# Assessment of flukicide efficacy against *Fasciola hepatica* in sheep in Sweden in the absence of a standardised test

**DOI:** 10.1016/j.ijpddr.2016.06.004

**Published:** 2016-06-26

**Authors:** Adam Novobilský, Natalia Amaya Solis, Moa Skarin, Johan Höglund

**Affiliations:** Department of Biomedical Sciences and Veterinary Public Health, Section for Parasitology, Swedish University of Agricultural Sciences (SLU), 75007, Uppsala, Sweden

**Keywords:** Albendazole, Coproantigen ELISA, Egg hatch test, Faecal egg count, In vitro, Liver fluke, Resistance

## Abstract

Anthelmintic resistance (AR) to *Fasciola hepatica* is emerging worldwide. Recently, AR to the adulticide compound albendazole (ABZ) was shown in Argentina and Spain. In Sweden, ABZ treatment failure against *F*. *hepatica* was first reported in sheep in 2012. The present study tested the efficacy of ABZ and triclabendazole (TCBZ) in sheep naturally infected with *F*. *hepatica* using a combination of three different diagnostic methods: faecal egg counts (FEC), coproantigen ELISA (cELISA) and *Fasciola* egg hatch test (FEHT). Two deworming trials, in November 2014 and January 2015, were performed on two sheep farms (farms A and B) in south-western Sweden. Except ABZ in November, treatment with ABZ or TCBZ achieved sufficient efficacy (97–100%) against adult *F*. *hepatica* on farm A. In contrast, ABZ treatment failed in the sheep flock on farm B, despite low initial faecal egg output. On farm B, ABZ efficacy based on FEC was 67% (95% CI: 35–84) and four of eight ewes tested were coproantigen-positive 21 days post-treatment. Ovicidal activity of ABZ against *Fasciola* eggs in isolates from both farms and one additional bovine isolate were tested by FEHT to exclude the presence of juvenile flukes and other factors such as dosing failure and poor quality of drug product. Irrespective of drug trial, data from FEHT showed significantly lower ovicidal activity of ABZ for the ovine farm B isolate than for the isolate from farm A. This confirms that the low efficacy of ABZ in sheep flock B was associated with ABZ resistance. Overall, the usefulness of three complementary methods for detection of ABZ resistance in the field was demonstrated.

## Highlights

• Efficacy of albendazole and triclabendazole against *Fasciola hepatica* was tested in sheep.
• Coproantigen ELISA, faecal egg counts and *in vitro Fasciola* egg hatch test were used.
• Albendazole resistance in *F*. *hepatica* was confirmed in one sheep flock.
• *Fasciola* egg hatch test is a useful tool for detection of albendazole resistance.

## Graphical abstract

**Figure:**
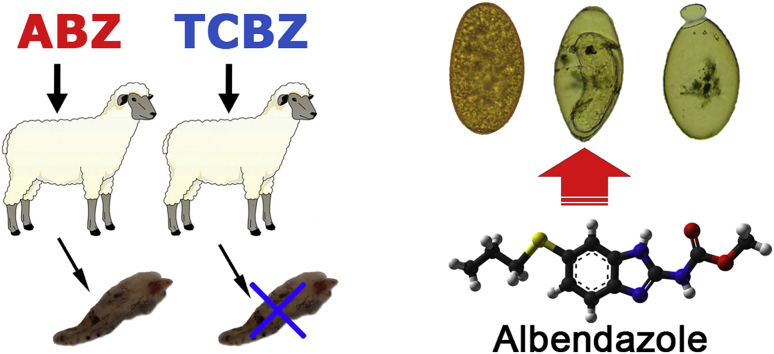

## 1 Introduction

The common liver fluke (*Fasciola hepatica*) causes significant production disease in sheep and cattle worldwide. Control of fasciolosis is based on treatment with flukicides that differ in chemical structure and mode of action, but also in their efficacy against different liver fluke developmental stages (Fairweather and Boray, 1999). Triclabendazole (TCBZ) and albendazole (ABZ) are common benzimidazoles used against liver fluke infection (Skuce and Zadoks, 2013). TCBZ is effective against both adult and juvenile flukes from the age of 2 days, and is the drug of choice for both sheep and cattle, whereas ABZ is effective only against adult flukes (from 12 weeks) (Fairweather and Boray, 1999). A demonstrated minimal inhibition concentration of ABZ for all animal products and a short withdrawal period for milk (60 h) (Power et al., 2013) make ABZ one of the most useful flukicides for dairy cattle.

Resistance of liver flukes to flukicides had not been noted until the first report of TCBZ resistance in Australia in 1995 (Overend and Bowen, 1995). Since then, numerous cases of TCBZ resistance have been reported in Australia (Brockwell et al., 2014), Europe (Moll et al., 2000, Daniel et al., 2012, Gordon et al., 2012a, Hanna et al., 2015) and South America (Olaechea et al., 2011). The significant increase in reported TCBZ resistance has raised new issues regarding evaluation of flukicide efficacy, diagnostic methods for anthelmintic resistance (AR) and their interpretation. There is no standard recommended protocol for determination of flukicide efficacy/resistance (Fairweather, 2011b, Fairweather et al., 2012). Faecal egg count reduction test (FECRT) (Coles et al., 2000, Moll et al., 2000, Hanna et al., 2015), coproantigen reduction test (CRT) (Flanagan et al., 2011a, Flanagan et al., 2011b, Brockwell et al., 2013), detection of fluke DNA in faeces by polymerase chain reaction (PCR) (Robles-Perez et al., 2013), post-mortem fluke counts (Coles and Stafford, 2001) and histology of flukes exposed *in vivo* (Hanna et al., 2011) have all been used for detection of flukicide efficacy in ruminants. In common veterinary practice, a combination of FECRT and CRT seems to be the most promising methods for detection (Gordon et al., 2012b, Novobilský et al., 2012, Brockwell et al., 2014, Elliott et al., 2015, Novobilský and Höglund, 2015). For most methods, however, specific thresholds have not yet been determined, which makes interpretation problematic, in particular as drug evaluation can be affected by several other factors such as incorrect dosing, metabolism status of animals treated, inferior quality of the flukicide product used and/or timing of the drug efficacy trial (Fairweather, 2011a). Altogether, this makes diagnosis of flukicide resistance complicated.

*In vitro* screening of AR is used for some gastrointestinal nematodes, but its application is limited for liver flukes. No reliable molecular markers for TCBZ resistance have so far been reported (Elliott and Spithill, 2014, Hodgkinson et al., 2013). However, in parallel with larval *in vitro* testing in nematodes, the *Fasciola* egg hatch test (FEHT) has been developed to diagnose susceptibility of *F. hepatica* eggs to ABZ (Alvarez et al., 2009, Canevari et al., 2014, Robles-Perez et al., 2014) and TCBZ (Fairweather et al., 2012, Robles-Perez et al., 2015).

In Sweden, control of bovine and ovine fasciolosis has been based solely on ABZ for decades (Novobilský et al., 2012, Novobilský et al., 2015b). Failure of ABZ treatment in sheep has been reported on a single farm in south-western Sweden (Novobilský et al., 2012). However, the exact reasons for this ABZ failure remain unknown. The presence of juvenile flukes that are unaffected by ABZ makes diagnosis of adulticide efficacy impractical, especially in cases where animals are kept outdoors all year round. Implementation of FEHT, which is independent of drug trial data, metabolism and re-infection status of animals tested, would improve diagnosis of resistance to adulticide anthelmintics such as ABZ. The aim of the present study was thus to evaluate ABZ and TCBZ efficacy in sheep naturally infected with *F*. *hepatica* using a combination of faecal egg counts (FEC) and coproantigen reduction, supported by FEHT. In addition, the effect of drug application timing was assessed.

## 2 Material and methods

### 2.1 Animals

Two flocks of Gotlandic Pelt sheep on islands of south-western Sweden with a long history of fasciolosis were selected for the study. Both farms are located off the west coast, north of Gothenburg, in a highly liver fluke abundant area (Novobilský et al., 2015a). One farm (farm A) is located on the island of Orust and the other (farm B) on the neighbouring island of Tjörn. The sheep flocks consist of 70 and 150 ewes on farm A and B, respectively. The distance between the farms is approx. 30 km, so the climatic conditions are almost identical at both locations. To control liver fluke, albendazole (ABZ) or/and triclabendazole (TCBZ) have been applied orally in both sheep flocks for a period of 10 years and 2 years, respectively. Animals were last treated (with ABZ) 12 months before the study. In order to select animals with patent liver fluke infection, 50% of the ewes in each flock were sampled in October 2014. In this pre-screening test, faecal samples were collected and examined for the presence of *F*. *hepatica* coproantigen as described below. Based on the results of pre-screening, coproantigen-positive ewes were randomly divided into groups of 6–8 animals.

### 2.2 Deworming and evaluation of drug efficacy

On both farms, two groups of 6–8 ewes were treated with ABZ or TCBZ in January (9 and 11 weeks after housing on farm A and B, respectively). To compare the efficacy of ABZ between autumn and winter deworming, other two groups of ewes were treated with ABZ or TCBZ on farm A in November (4 weeks after housing). However, November deworming was not feasible on Farm B, as the ewes were already pregnant at that time and the use of ABZ is contraindicated in such cases. All animals were weighed and treated orally with a commercial formulation of either ABZ (Valbazen^®^, Pfizer) at a dose of 7.6 mg per kg bodyweight or TCBZ (Triclafas^®^ Oral Drench, Norbrook) at a dose of 10 mg per kg bodyweight. The drug was administered in 50 ml syringes with 0.2 ml accuracy by a licensed veterinary surgeon (employed by Farm & Animal Health, Sweden) as part of routine deworming. No ethical permission was required according to the Animal Welfare Act 2009/021. All drugs were stored in a cool, dry place until use. The animals were kept indoors during the entire study.

### 2.3 Faecal egg counts and coproantigen ELISA

Faecal samples were collected on days 0, 7 and 21 post-treatment, based on previous studies (Moll et al., 2000, Brockwell et al., 2014, Hanna et al., 2015). The samples were labelled with the animal ID on ear tags and immediately sent by mail to our laboratory in Uppsala. The samples were stored at 4 °C during delivery and storage and processed immediately after arrival. The maximum interval between sampling on the farm and sample processing in the laboratory was 48 h. The FEC were conducted by the sedimentation method on 3 g of faeces as described by Novobilský and Höglund (2015). Coproantigen in faecal samples was determined using a commercial kit BIO-X ELISA (BIO K 201, BIO-X Diagnostics, Belgium) according to the manufacturer’s protocol, where coproantigen levels are expressed as a percentage of positivity of optical density of sample versus optical density of a positive reference sample. The cut-off value for cELISA was optimised by previous examination of truly *F*. *hepatica*-positive and -negative sheep faecal samples (Brockwell et al., 2013, Novobilský and Höglund, 2015). Reference positive sheep faecal samples originated from previous studies (Novobilský et al., 2012, Novobilský et al., 2014) where infection status was confirmed by coproscopy, serology and liver fluke burden. Negative sheep samples came from *F*. *hepatica*-free herds in Uppsala.

### 2.4 *In vitro Fasciola* egg hatch test

Sensitivity of the two ovine *F*. *hepatica* isolates to ABZ was assessed by *in vitro* FEHT. The principles of FEHT have been described elsewhere (Alvarez et al., 2009, Fairweather et al., 2012, Canevari et al., 2014), although our protocol was somewhat modified as described below. Eggs were obtained from pooled faecal samples before treatment in both herds (farms A and B). An additional cattle *F*. *hepatica* isolate was obtained from adult worms collected at a local abattoir (Varekils Slakteri, Lundby) from the liver of beef cattle from farm C, located approximately 5 km from sheep farm B.

The FEHT was carried out in 24-well, flat-bottomed plastic plates (Nunc, Sigma-Aldrich, Sweden). Albendazole powder (Sigma-Aldrich, Sweden; product ID A4673) was dissolved in maximum 0.5% dimethyl sulphoxide (DMSO) (Sigma Aldrich, Sweden, product ID 472301). This maximum concentration of DMSO had no effect on egg development in our preliminary tests (Amaya-Solis, 2012) or in a previous study (Fairweather et al., 2012). Fluke eggs were exposed to ABZ at a final concentration of 0.02, 0.1, 0.5, 2.5, 12.5 nmol/ml and 0.5% DMSO only (negative control) for 12 h at 25 °C. Each concentration was tested in four replicates containing approximately 100 eggs per well. After exposure, the eggs were washed three times in tap water and then incubated in 24-well plates in 2 ml tap water for 14 days at 25 °C in the dark. After incubation, miracidial hatching was stimulated by exposure to intensive light for 12 h. Hatching was terminated by adding of 0.1 ml Lugol’s iodine solution to each well 4 h after exposure to light. Hatched and unhatched, including dead eggs and eggs with developed miracidia were counted under an inverted microscope at 40x magnification. Ovicidal activity of ABZ was calculated according to the formula, below: Ovicidal activity (%) = (% of eggs hatched in negative control - % eggs hatched after drug exposure/% of eggs hatched in negative control)*100 (Alvarez et al., 2009, Canevari et al., 2014).

### 2.5 Statistical analysis

Faecal egg count was calculated by two independent methods. First, FEC reduction was expressed as a comparison of arithmetic means between pre-treatment and post-treatment levels with 95% confidence limit, as recommended by The World Association for the Advancement of Veterinary Parasitology (WAAVP) (Coles et al., 1992). In the second method, the percentage of FEC reduction was obtained using the online Bayesian hierarchical modelling platform “eggCounts” http://www.math.uzh.ch/as/index.php?id=254 (Torgerson et al., 2014).

The cut-off value for coproantigen ELISA was calculated using receiver operating characteristic (ROC) analysis. Treatment effect between drugs and also between deworming periods in each herd was assessed by two-way ANOVA, followed by Bonferroni post-tests with significance *P* < 0.05. One-way analysis of variance (ANOVA) was applied to evaluate differences between pre-treatment and post-treatment FEC and coproantigen levels in each group. In FEHT, ABZ concentration was plotted against percentage of ovicidal activity after probit transformation. A non-linear regression model was then built to fit dose-response data from FEHT. The EC_50_ values, 95% confidence intervals and R^2^ values were calculated for each *F*. *hepatica* isolate. All analyses were conducted using Graph Pad Prism 5.02 (GraphPad Software, USA).

## 3 Results

**Fig. 1:**
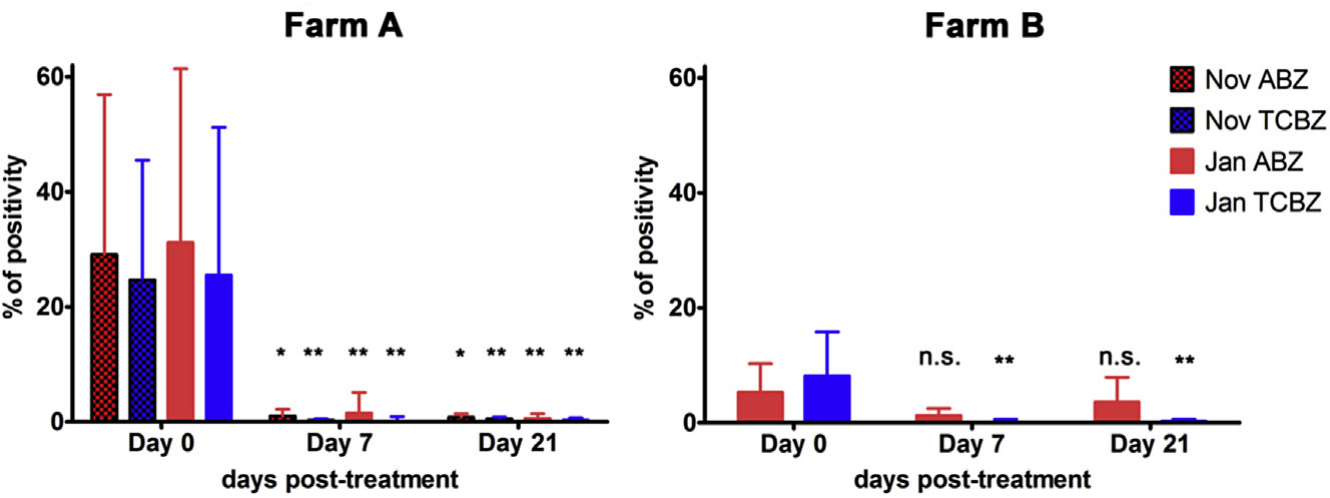
Mean coproantigen values for sheep treated with albendazole (ABZ, 7.6 mg/kg) and triclabendazole (TCBZ, 10 mg/kg) on farm A (Orust) and farm B (Tjörn), in faecal samples obtained at days 0, 7 and 21 days post-treatment for deworming in November 2014 and January 2015. Coproantigen values are expressed as % of positivity. Abbreviations: *Indicates significant difference (*P < 0.05; **P < 0.01) between pre-treatment and post-treatment time interval. n.s. = not significant.

The cut-off value for coproantigen ELISA based on 40 positive and 40 negative control samples was 1.6% positivity. TCBZ was highly efficient (FECR 97–100%) in both flocks, irrespective of deworming period, as documented by FEC and coproantigen levels (Fig. 1, Table 1, Table 2). On farm A, ABZ efficacy based on FEC (21 days post-treatment) was slightly lower for November treatment (92%) than for January treatment (99%), but this difference was not significant. FECR values were lower on day 7 in both ABZ and TCBZ groups and raised to 99% on day 21 post-treatment (Table 2) on farm A likely due to continuous release of eggs remaining in bile ducts and gall bladder. Coproantigen efficacy of ABZ was similar in both the November and January treatments on day 21 post-treatment. In addition, no significant difference in FEC and coproantigen (*P* > 0.05) was observed between November and January treatment for the TCBZ and ABZ groups on farm A. On farm B, both coproantigen and FEC data showed low efficacy in the ABZ group, where 50% of animals remained coproantigen-positive 21 days post-treatment (Table 1, Table 2, Fig. 1).

**Fig. 2:**
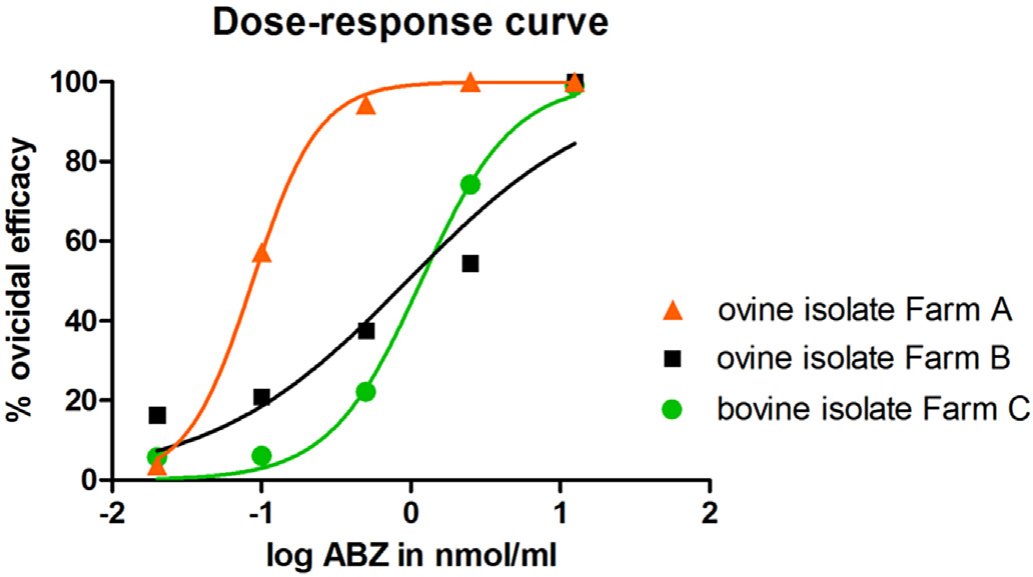
Dose-response curves of albendazole (ABZ) ovicidal activity for ovine (farms A and B) and bovine (farm C) *Fasciola hepatica* egg isolates after probit transformation of ABZ concentrations.

The FEHT data showed an obvious difference in sensitivity to ABZ between *F*. *hepatica* eggs isolated from animals on farms A and B. At a concentration of 2.5 nmol/ml, the ovicidal activity of ABZ was 100% for the ovine isolate from farm A but only 54% for the farm B isolate. Furthermore, the bovine isolate from farm C displayed similar poor ovicidal activity to the ovine isolate from farm B. The EC_50_ values for the ovine and bovine isolates from farms B and C were approximately 10-fold higher than that obtained for the ovine isolate from farm A. Data from FEHT, including EC_50_ and R^2^ values, are summarised in Table 3 and Fig. 2.

## 4 Discussion

It has been suggested that ABZ resistance may be present in *F*. *hepatica* in Swedish sheep flocks in response to long term use of lower doses (e.g. 5 mg/kg bodyweight) than the recommended (7.5 mg/kg) in most countries (Novobilský et al., 2012). However, until now, convincing evidence for this claim has been lacking. In the present study, both reduced efficacy and decreased sensitivity of fluke eggs to ABZ was confirmed in a *F*. *hepatica* population from one sheep flock on the Swedish west coast using two independent coprological methods and an *in vitro* egg hatch test, respectively. ABZ resistance is difficult to prove in the field due to the potential presence of juvenile flukes, which are tolerant to the drug and thus unaffected by treatment. However, as the results of the *in vitro* test confirmed the outcome from the field trial, the observed ABZ treatment failure was most likely associated with resistance.

When flukicides are applied in general practice, diagnosis of flukicide efficacy/resistance can be compromised by several factors. For example, underdosing, poor quality of anthelmintic product, age of product, changes in drug metabolism within animals, inappropriate diagnostic tests may often be misdiagnosed as AR (Fairweather, 2011a, Skuce and Zadoks, 2013). In addition, in the case of adulticide drugs, misinterpretation related to the development of juvenile flukes surviving treatment must always be considered (Coles et al., 2006). The WAAVP guidelines cite dose and slaughter trials as the only definitive method for detection of AR (Coles et al., 2006). However, this involves costly and lengthy experimental infection of cultured snails and maintenance of experimental animals. Implementation of FEHT in diagnosing flukicide resistance provides a new approach that is independent of drug efficacy trials in treated animals. In the present study, we observed ABZ failure on farm B, as documented by FEC (67% efficacy) and coproantigen (32% efficacy) (Table 2). However, it should be emphasised that ABZ was applied in the beginning of January, exactly 9 weeks after housing on this farm. Thus, presence of juvenile flukes cannot be completely excluded on farm B and a decreasing trend in efficacy of both FEC and coproantigen (Table 2) could hypothetically indicate maturation of juvenile flukes surviving treatment. Furthermore, both initial FEC and coproantigen levels were very low, suggesting a modest fluke burden. In nematodes, low initial FEC before treatment decreases the reliability of FECRT (Coles et al., 1992). It can be assumed that a similar pattern applies for FEC and coproantigen in *F*. *hepatica*. Thus, decisions about the resistance status of the ovine *F*. *hepatica* population on farm B cannot be completely conclusive based only on the FECR and cELISA results. On the other hand, significant differences in ovicidal activity in FEHT between the ovine isolates studied here confirmed that the isolate from farm B possessed significantly lower susceptibility to ABZ than the isolate from farm A. Obvious differences between the isolates studied were also observed in terms of ABZ efficacy based on FEC and cELISA. Thus, the *F*. *hepatica* isolate from farm B is most likely resistant to ABZ.

The majority of studies dealing with flukicide resistance focus on the most effective drug, TCBZ, which is effective against both juvenile and adult flukes in livestock (see review by Fairweather, 2011b). In contrast, diagnosis of ABZ susceptibility/resistance has been neglected. So far, the only experimentally confirmed published account of a “dose and slaughter trial” ABZ-resistant fluke population is in Argentina (Sanabria et al., 2013). Field-based cases of ABZ resistance have been reported in Spain and Egypt (Alvarez-Sanchez et al., 2006, Robles-Perez et al., 2013, Shokier et al., 2013, Martinez-Valladares et al., 2014). In addition, the Argentinian CEDIVE isolate (Sanabria et al., 2013) and the Spanish Santillán de la Vega isolate (Robles-Perez et al., 2013) have been confirmed as ABZ-resistant by *in vitro* FEHT (Canevari et al., 2014, Robles-Perez et al., 2014). However, reports of field-documented flukicide resistance that subsequently turn out as incorrect by further investigation have also been presented (Fairweather, 2011b). This shows that combining field drug trials with *in vitro* FEHT is necessary for correct diagnosis of ABZ resistance. Interestingly, ABZ is effective in TCBZ-resistant populations (Coles and Stafford, 2001) despite both actives being benzimidazole derivates. At the same time sheep with ABZ-resistant flukes have been successfully treated with TCBZ (Robles-Perez et al., 2013, Sanabria et al., 2013), as also documented in our study. This clearly supports the theory that the molecular mechanisms of ABZ and TCBZ resistance differ.

Underdosing, long-term use of compounds with the same mode of action and high frequency of treatment are key factors in development of AR in gastrointestinal nematodes (Kaplan, 2004). In Sweden, ABZ has been used against liver flukes for decades, as until recently it was the only licensed flukicide. However, the dose used for deworming is 1.5-fold lower than recommended in several other countries (Novobilský et al., 2012). Therefore, the recommended dose of Valbazen (ABZ) in Sweden was recently increased to 7.6 mg/kg of ABZ, i.e. the dose that was used in this study. Repeated underdosing and long-term use of ABZ only might explain the emergence of ABZ resistance in Sweden.

Albendazole acts only against mature flukes older than 12 weeks post-infection (Johns and Dickeson, 1979, Coles and Stafford, 2001). Thus, ABZ is not usually effective when applied during or immediately after the grazing season, due to the presence of immature flukes (Mitchell, 2002). Surprisingly, the final effect of drug application timing between November and January deworming in this study did not differ for either drug on farm A. Although FECR for ABZ was slightly lower in November, coproantigen values did not differ from the January results. In the autumn treatment, the drugs were applied 4 weeks after housing. Although immature flukes could be expected in ewes at that time of the year, ABZ efficacy was similar to that observed at the January treatment suggesting that all flukes were mature already in November. In Sweden, sheep are infected at the earliest in May–June with metacercarial cysts shed from overwintering snails, but also in July–August as a result of summer infection (Novobilský et al., 2014). Thus, sheep on farm A were most likely infected in early summer and, from a practical point of view, our results indicate that treatment with ABZ could be applied from November onwards during the housing period.

The FEHT is a relatively new *in vitro* method for testing the ovicidal activity of flukicides. In this study, we used short-term exposure (12 h) to ABZ according to Alvarez et al. (2009), as this time corresponds to *in vivo* exposure. In other studies (Fairweather et al., 2012, Robles-Perez et al., 2014, Robles-Perez et al., 2015), *Fasciola* eggs were incubated for 14 days until hatching of miracidia. Using DMSO at a final concentration of 0.5% in our protocol did not affect the viability of the eggs. This concentration was suggested by our preliminary testing (Amaya-Solis, 2012), and by a previous *in vitro* study with TCBZ (Fairweather et al., 2012). Higher concentrations of DMSO seem to have an ovicidal effect in *F*. *hepatica*.

In FEHT optimisation, the highest variation in the ovicidal activity of ABZ was observed in the range 0.1–2.5 nmol/ml (Amaya-Solis, 2012). Similarly, Canevari et al. (2014) showed that 0.5 nmol/ml is the threshold ABZ concentration to discriminate between ABZ-resistant and ABZ-susceptible *F*. *hepatica* isolates. At this ABZ concentration, ovicidal efficacy of ≤40% or ≥70% indicates resistance and susceptibility to ABZ, respectively. We observed ovicidal efficacy of 94% (farm A), 22% (farm B) and 37% (farm C), suggesting that the ovine and bovine isolates from two nearby farms (B and C, respectively) were most likely ABZ-resistant. In nematodes, susceptibility/resistance status of isolates in *in vitro* tests is compared strictly on a dose-response basis using linear or non-linear regression and comparison of data against a standard resistant reference strain (Taylor et al., 2002, Demeler et al., 2010). In this study, *F*. *hepatica* isolates were exposed to ABZ concentrations in the range 0.02–12.5 nmol/ml and there was a noticeable dose-response effect. High R^2^ values (R^2^ > 0.9 for all three isolates) in non-linear regression showed that the model fitted our data. Since no standard ABZ-resistant reference *F*. *hepatica* isolate is available, interpretation of FEHT data is difficult. However, the EC_50_ value of the ovine isolate from farm A was 10-fold lower than that obtained for the ovine (farm B) and bovine (farm C) isolates from Tjörn, suggesting significant differences in their susceptibility to ABZ.

## 5 Conclusions

Albendazole is a commonly used flukicide around the world. Unlike in many other European countries it has long been used as drug of first choice against liver flukes in sheep in Sweden. The first confirmed case of ABZ resistance in sheep in Sweden is reported in this study. This finding should be taken into account in future planning of control strategies against fasciolosis. Combined testing with FEC, cELISA and FEHT appears to be efficient in diagnosis of ABZ resistance in the field. Despite high efficacy of ABZ at November deworming observed in the study, we recommend using ABZ at least 12 weeks after housing in sheep, since time of fluke infection may vary between locations. In addition, ABZ is contraindicated in the first 30 days of gestation.

## Conflict of interest statement

The authors declare that they have no competing interests.

## Figures and Tables

**Table 1:** Summary of faecal egg counts (FEC) expressed as eggs per gram faeces (EPG) and coproantigen level (% positivity) measured on sheep farms on farm A (Orust) and farm B (Tjörn).

		Farm A								Farm B			
		November				January				January			
		Day -26	Day 0	Day 7	Day 21	Day -75	Day 0	Day 7	Day 21	Day -61	Day 0	Day 7	Day 21
ABZ	Mean FEC (EPG) ± standard deviation	n.a.	56.0 ± 44.1	0.7 ± 1.2	4.6 ± 10.5	n.a.	135.5 ± 153.0	60.3 ± 131.4	1.8 ± 4.5	n.a.	3.9 ± 4.6	1.1 ± 2.2	1.2 ± 1.4
	Number of F. hepatica egg shedding animals (number of animals in the group)	n.a.	6 (6)	3 (6)	3 (6)	n.a.	8 (8)	3 (8)	3 (8)	n.a.	8 (8)	4 (8)	5 (8)
	Mean % coproantigen positivity ± standard deviation	41.9 ± 34.9	29.1 ± 27.8	1.0 ± 1.2	0.8 ± 0.6	29.0 ± 25.8	31.2 ± 30.2	1.5 ± 3.6	0.6 ± 0.8	8.1 ± 9.0	5.3 ± 5.0	1.3 ± 1.2	3.6 ± 4.3
	Number of coproantigen positive animals (number of animals in the group)	6 (6)	6 (6)	2 (6)	0 (6)	6 (8)^a^	8 (8)	1 (8)	1 (8)	8 (8)	6 (8)	3 (8)	4 (8)
TCBZ	Mean FEC (EPG) ± standard deviation	n.a.	72.8 ± 71.2	13.4 ± 30.8	0.1 ± 0.1	n.a.	62.9 ± 76.7	9.6 ± 14.3	0.4 ± 0.3	n.a.	3.1 ± 2.1	0.0	0.0
	Number of F. hepatica egg shedding animals (number of animals in the group)	n.a.	8 (8)	5 (8)	2 (8)	n.a.	7 (7)	3 (7)	1 (7)	n.a.	7 (7)	0 (7)	0 (7)
	Mean % coproantigen positivity ± standard deviation	36.5 ± 27.7	24.7 ± 20.8	0.3 ± 0.2	0.5 ± 0.3	37.8 ± 25.6	25.5 ± 25.7	0.1 ± 0.8	0.3 ± 0.4	7.8 ± 7.8	8.2 ± 7.6	0.1 ± 0.5	0.3 ± 0.3
	Number of coproantigen positive animals (number of animals in the group)	8 (8)	8 (8)	0 (8)	0 (8)	6 (7)^a^	7 (7)	0 (7)	0 (7)	7 (7)	6 (7)	0 (7)	0 (7)

Abbreviations:ABZ = albendazole, TCBZ = triclabendazole.

a On farm A in January, 2 and 1 sheep were slaughtered in period between pre-screening (Day -75) and Day 0 in ABZ and TCBZ group, respectively. These slaughtered animals were replaced with 3 other coproantigen and *Fasciola hepatica* egg positive sheep on Day 0. Data for these 3 slaughtered sheep were not included on Day -75.

**Table 2:** Efficacy of albendazole and triclabendazole against liver flukes based on faecal egg counts (FEC) and coproantigen ELISA tests on faecal samples obtained 7 and 21 days post-treatment on sheep farms on Orust (farm A) and Tjörn (farm B).

		Farm A				Farm B	
		November		January		January	
		Day 7	Day 21	Day 7	Day 21	Day 7	Day 21
Albendazole	FECR in %, (95% CI)^a^	99 (94–100)	92 (35–99)	56 (0–93)	99 (90–100)	71 (0–95)	69 (0–91)
	FECR in %, (95% CI)^b^	99 (97–100)	92 (88–94)	56 (51–60)	99 (98–99)	73 (45–88)	67 (35–84)
	Coproantigen reduction in %	97	97	95	98	76	32
Triclabendazole	FECR in %, (95% CI)^a^	82 (0–97)	100	85 (30–97)	99 (94–100)	100	100
	FECR in %, (95% CI)^b^	82 (77–85)	100 (99–100)	85 (80–88)	99 (98–100)	97 (82–100)	97 (83–100)
	Coproantigen reduction in %	99	98	100	99	99	97

Abbreviations: 95% CI = 95% confidence interval, FECR = faecal egg count reduction.

a Faecal egg count reduction calculated according to Coles et al. (1992).

b Faecal egg count reduction calculated according to Torgerson et al. (2014).

**Table 3:** Ovicidal efficacy determined by *in vitro Fasciola* egg hatch test (FEHT) of albendazole (ABZ) at concentrations 0.02, 0.1, 0.5, 2.5, and 12.5 nmol/ml against *F*. *hepatica* eggs of two ovine isolates (farm A and B) and one bovine isolate (farm C).

ABZ (nmol/ml)	Ovicidal efficacy		
	Ovine isolate (Farm A)	Ovine isolate (Farm B)	Bovine isolate (Farm C)
12.5	100	100	100
2.5	100	54.5	74.2
0.5	94.4	37.5	22.2
0.1	57.2	20.9	6.1
0.02	3.6	16.4	5.8
EC_50_	0.087	0.947	1.171
95% CI of EC_50_	0.078 to 0.098	0.207 to 4.334	0.822 to 1.667
R^2^	0.998	0.901	0.993

Abbreviations:; EC_50_ = effective concentration for 50% inhibition; R^2^ = coefficient in regression analysis.

## References

[bib1] Alvarez L., Moreno G., Moreno L., Ceballos L., Shaw L., Fairweather I., Lanusse C. (2009). Comparative assessment of albendazole and triclabendazole ovicidal activity on *Fasciola hepatica* eggs. Vet. Parasitol..

[bib2] Alvarez-Sanchez M.A., Mainar-Jaime R.C., Perez-Garcia J., Rojo-Vazquez F.A. (2006). Resistance of *Fasciola hepatica* to triclabendazole and albendazole in sheep in Spain. Vet. Rec..

[bib3] Amaya-Solis N. (2012). Development of an in vitro Egg Hatch Test to Diagnose Anthelmintic Resistance in *Fasciola Hepatica* in Sweden.

[bib4] Brockwell Y.M., Elliott T.P., Anderson G.R., Stanton R., Spithill T.W., Sangster N.C. (2014). Confirmation of *Fasciola hepatica* resistant to triclabendazole in naturally infected Australian beef and dairy cattle. Int. J. Parasitol. Drugs Drug Resist.

[bib5] Brockwell Y.M., Spithill T.W., Anderson G.R., Grillo V., Sangster N.C. (2013). Comparative kinetics of serological and coproantigen ELISA and faecal egg count in cattle experimentally infected with *Fasciola hepatica* and following treatment with triclabendazole. Vet. Parasitol..

[bib6] Canevari J., Ceballos L., Sanabria R., Romero J., Olaechea F., Ortiz P., Cabrera M., Gayo V., Fairweather I., Lanusse C., Alvarez L. (2014). Testing albendazole resistance in *Fasciola hepatica*: validation of an egg hatch test with isolates from South America and the United Kingdom. J. Helminthol..

[bib7] Coles G.C., Bauer C., Borgsteede F.H.M., Geerts S., Klei T.R., Taylor M.A., Waller P.J. (1992). World Association for the Advancement of Veterinary Parasitology (WAAVP) methods for the detection of anthelmintic resistance in nematodes of veterinary importance. Vet. Parasitol..

[bib8] Coles G.C., Jackson F., Pomroy W.E., Prichard R.K., von Samson-Himmelstjerna G., Silvestre A., Taylor M.A., Vercruysse J. (2006). The detection of anthelmintic resistance in nematodes of veterinary importance. Vet. Parasitol..

[bib9] Coles G.C., Rhodes A.C., Stafford K.A. (2000). Activity of closantel against adult triclabendazole-resistant *Fasciola hepatica*. Vet. Rec..

[bib10] Coles G.C., Stafford K.A. (2001). Activity of oxyclozanide, nitroxynil, clorsulon and albendazole against adult triclabendazole-resistant *Fasciola hepatica*. Vet. Rec..

[bib11] Daniel R., van Dijk J., Jenkins T., Akca A., Mearns R., Williams D.J.L. (2012). A composite faecal egg count reduction test to detect resistance to triclabendazole in *Fasciola hepatica*. Vet. Rec..

[bib12] Demeler J., Kuttler U., El-Abdellati A., Stafford K., Rydzik A., Varady M., Kenyon F., Coles G., Höglund J., Jackson F., Vercruysse J., von Samson-Himmelstjerna G. (2010). Standardization of the larval migration inhibition test for the detection of resistance to ivermectin in gastro-intestinal nematodes of ruminants. Vet. Parasitol..

[bib13] Elliott T.P., Kelley J.M., Rawlin G., Spithill T.W. (2015). High prevalence of fasciolosis and evaluation of drug efficacy against *Fasciola hepatica* in dairy cattle in the Maffra and Bairnsdale districts of Gippsland, Victoria, Australia. Vet. Parasitol..

[bib14] Elliott T.P., Spithill T.W. (2014). The T687G SNP in a P-glycoprotein gene of *Fasciola hepatica* is not associated with resistance to triclabendazole in two resistant Australian populations. Mol. Biochem. Parasit..

[bib15] Fairweather I. (2011). Raising the bar on reporting ’triclabendazole resistance’. Vet. Rec..

[bib16] Fairweather I. (2011). Reducing the future threat from (liver) fluke: realistic prospect or quixotic fantasy?. Vet. Parasitol..

[bib17] Fairweather I., Boray J.C. (1999). Fasciolicides: efficacy, actions, resistance and its management. Vet. J..

[bib18] Fairweather I., McShane D.D., Shaw L., Ellison S.E., O’Hagan N.T., York E.A., Trudgett A., Brennan G.P. (2012). Development of an egg hatch assay for the diagnosis of triclabendazole resistance in *Fasciola hepatica*: proof of concept. Vet. Parasitol..

[bib19] Flanagan A.M., Edgar H.W.J., Forster F., Gordon A., Hanna R.E.B., McCoy M., Brennan G.P., Fairweather I. (2011). Standardisation of a coproantigen reduction test (CRT) protocol for the diagnosis of resistance to triclabendazole in *Fasciola hepatica*. Vet. Parasitol..

[bib20] Flanagan A., Edgar H.W.J., Gordon A., Hanna R.E.B., Brennan G.P., Fairweather I. (2011). Comparison of two assays, a faecal egg count reduction test (FECRT) and a coproantigen reduction test (CRT), for the diagnosis of resistance to triclabendazole in *Fasciola hepatica* in sheep. Vet. Parasitol..

[bib21] Gordon D., Zadoks R., Skuce P., Sargison N. (2012). Confirmation of triclabendazole resistance in liver fluke in the UK. Vet. Rec..

[bib22] Gordon D.K., Zadoks R.N., Stevenson H., Sargison N.D., Skuce P.J. (2012). On farm evaluation of the coproantigen ELISA and coproantigen reduction test in Scottish sheep naturally infected with *Fasciola hepatica*. Vet. Parasitol..

[bib23] Hanna R.E., McMahon C., Ellison S., Edgar H.W., Kajugu P.E., Gordon A., Irwin D., Barley J.P., Malone F.E., Brennan G.P., Fairweather I. (2015). *Fasciola hepatica*: a comparative survey of adult fluke resistance to triclabendazole, nitroxynil and closantel on selected upland and lowland sheep farms in Northern Ireland using faecal egg counting, coproantigen ELISA testing and fluke histology. Vet. Parasitol..

[bib24] Hanna R.E.B., Gordon A.W., Moffett D., Edgar H.W.J., Oliver L.F., McConnell S., Shaw L., Brennan G.P., Fairweather I. (2011). *Fasciola hepatica*: comparative effects of host resistance and parasite intra-specific interactions on size and reproductive histology in flukes from rats infected with isolates differing in triclabendazole sensitivity. Vet. Parasitol..

[bib25] Hodgkinson J., Cwiklinski K., Beesley N.J., Paterson S., Williams D.J.L. (2013). Identification of putative markers of triclabendazole resistance by a genome-wide analysis of genetically recombinant *Fasciola hepatica*. Parasitology.

[bib26] Johns D.R., Dickeson S.J. (1979). Efficacy of albendazole against *Fasciola hepatica* in sheep. Aust. Vet. J..

[bib27] Kaplan R.M. (2004). Drug resistance in nematodes of veterinary importance: a status report. Trends Parasitol..

[bib28] Martinez-Valladares M., Cordero-Perez C., Rojo-Vazquez F.A. (2014). Efficacy of an anthelmintic combination in sheep infected with *Fasciola hepatica* resistant to albendazole and clorsulon. Exp. Parasitol..

[bib29] Mitchell G. (2002). Update on fasciolosis in cattle and sheep. In Pract..

[bib30] Moll L., Gaasenbeek C.P.H., Vellema P., Borgsteede F.H.M. (2000). Resistance of *Fasciola hepatica* against triclabendazole in cattle and sheep in the Netherlands. Vet. Parasitol..

[bib31] Novobilský A., Averpil H.B., Höglund J. (2012). The field evaluation of albendazole and triclabendazole efficacy against *Fasciola hepatica* by coproantigen ELISA in naturally infected sheep. Vet. Parasitol..

[bib32] Novobilský A., Engström A., Sollenberg S., Gustafsson K., Morrison D.A., Höglund J. (2014). Transmission patterns of *Fasciola hepatica* to ruminants in Sweden. Vet. Parasitol..

[bib33] Novobilský A., Höglund J. (2015). First report of closantel treatment failure against *Fasciola hepatica* in cattle. Int. J. Parasitol. Drugs Drug Resist.

[bib34] Novobilský A., Novák J., Björkman C., Höglund J. (2015). Impact of meteorological and environmental factors on the spatial distribution of *Fasciola hepatica* in beef cattle herds in Sweden. BMC Vet. Res..

[bib35] Novobilský A., Sollenberg S., Höglund J. (2015). Distribution of *Fasciola hepatica* in Swedish dairy cattle and associations with pasture management factors. Geospat. Health.

[bib36] Olaechea F., Lovera V., Larroza M., Raffo F., Cabrera R. (2011). Resistance of *Fasciola hepatica* against triclabendazole in cattle in Patagonia (Argentina). Vet. Parasitol..

[bib37] Overend D.J., Bowen F.L. (1995). Resistance of *Fasciola hepatica* to triclabendazole. Aust. Vet. J..

[bib38] Power C., Sayers R., O’Brien B., Furey A., Danaher M., Jordan K. (2013). Review of studies on flukicide residues in cows’ milk and their transfer to dairy products. Ir. J. Agr. Food. Res..

[bib39] Robles-Perez D., Martinez-Perez J.M., Rojo-Vazquez F.A., Martinez-Valladares M. (2013). The diagnosis of fasciolosis in feces of sheep by means of a PCR and its application in the detection of anthelmintic resistance in sheep flocks naturally infected. Vet. Parasitol..

[bib40] Robles-Perez D., Martinez-Perez J.M., Rojo-Vazquez F.A., Martinez-Valladares M. (2014). Development of an egg hatch assay for the detection of anthelmintic resistance to albendazole in *Fasciola hepatica* isolated from sheep. Vet. Parasitol..

[bib41] Robles-Perez D., Martinez-Perez J.M., Rojo-Vazquez F.A., Martinez-Valladares M. (2015). Screening anthelmintic resistance to triclabendazole in *Fasciola hepatica* isolated from sheep by means of an egg hatch assay. BMC Vet. Res..

[bib42] Sanabria R., Ceballos L., Moreno L., Romero J., Lanusse C., Alvarez L. (2013). Identification of a field isolate of *Fasciola hepatica* resistant to albendazole and susceptible to triclabendazole. Vet. Parasitol..

[bib43] Shokier K.M., Aboelhadid S.M., Waleed M.A. (2013). Efficacy of five anthelmintics against a natural *Fasciola* species infection in cattle. Beni-Suef Univ. J. Bas. App. Sci..

[bib44] Skuce P.J., Zadoks R.N. (2013). Liver fluke: a growing threat to UK livestock production. Cattle Pract..

[bib45] Taylor M.A., Hunt K.R., Goodyear K.L. (2002). Anthelmintic resistance detection methods. Vet. Parasitol..

[bib46] Torgerson P.R., Paul M., Furrer R. (2014). Evaluating faecal egg count reduction using a specifically designed package “eggCounts” in R and a user friendly web interface. Int. J. Parasitol..

